# Cervical spine giant cell bone tumor: a case report

**DOI:** 10.1186/s12957-019-1625-5

**Published:** 2019-05-11

**Authors:** İdris Sertbaş, Mete Karatay, Uguray Payam Hacisalihoğlu

**Affiliations:** 1 Department of Neurosurgery, Yeni Yüzyıl University Medical Faculty, Merkez Mahallesi,Çukurçeşme Caddesi No:51, Gaziosmanpaşa, İstanbul, Turkey; 2 Department of Pathology, Yeni Yüzyıl University Medical Faculty, Merkez Mahallesi, Çukurçeşme Caddesi No:51, Gaziosmanpaşa, İstanbul, Turkey

**Keywords:** Giant cell tumor, Cervical spine, Corpectomy

## Abstract

**Background:**

Giant cell tumors (GCTs) of the bone are locally aggressive primary bone tumors with a benign character. Spinal involvement is rare and they are quite rare in the cervical spine.

**Case presentation:**

A 31-year-old male patient presented with neck pain. Cervical CT revealed a lytic lesion extending posteriorly and causing the collapse of the C4 vertebra corpus. The patient underwent excision of the tumor extending from the anterior to the posterior with a single-stage anterior intervention followed by the placement of an anterior cage and plate-screw system for fusion. The pathology was reported as GCT.

**Conclusions:**

The posteriorly located lesion was widely curetted through an anterior approach in a single session.

## Background

Giant cell tumors (GCTs) of the bone are locally aggressive primary tumors with a benign character involving the metaphysis of long bones. The tumor develops after physeal closure and causes pathological fractures. The incidence in the spinal cord varies between 1.4% and 9.4% and the most common spinal location is the sacrum. The incidence in the cervical spine is quite low [1, 2]. Primary GCTs in cervical spine constitute 2–3% of all spinal tumors [3, 4]. It is more common in females than males and in the third and fourth decades. The symptom is tenderness in the tumor region. Vertebral GCTs can cause a neurological deficit by growing inside and compressing the spinal canal [5]. Giant cell tumors of the bone are radiologically osteolytic and destructive lesions. The preferred treatment for GCTs is wide en bloc resection but spinal GCTs may not be able to be resected en bloc due to the risk of vascular or neural injury [6, 7]. Common treatment option for spinal GCTs is curettage but if the tumor is removed incompletely, local recurrence and/or metastasis is usually seen [8].

## Case report

**Fig. 1:**
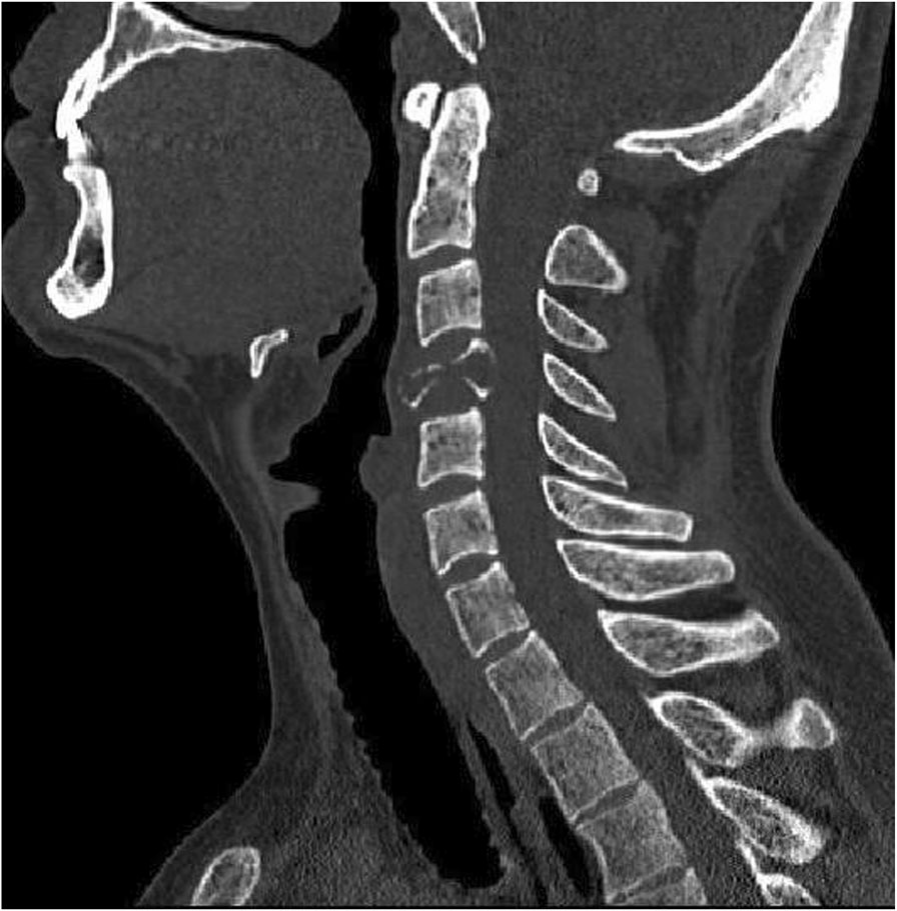
Lytic lesion in C4 vertebral corpus on CT in the sagittal plane

**Fig. 2:**
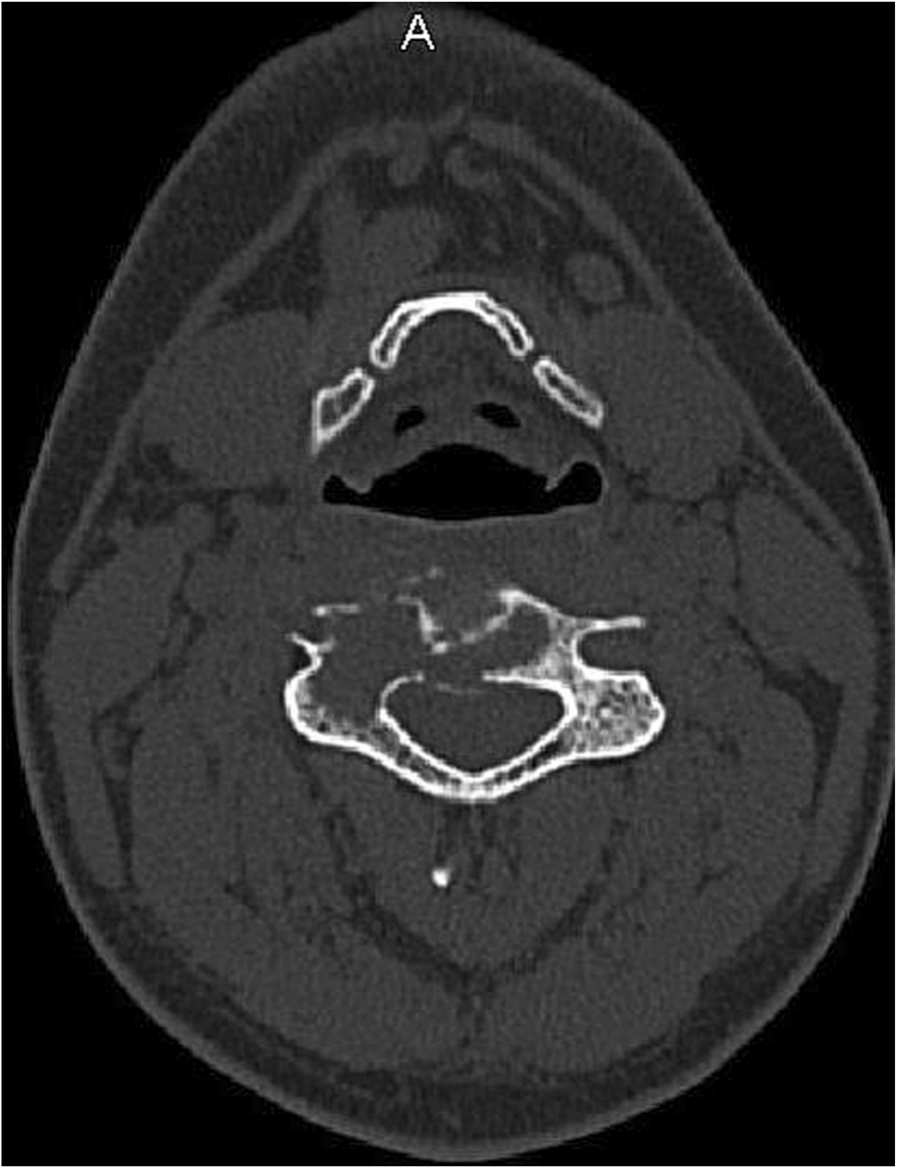
Lesion extending from the vertebra corpus to the lateral mass on CT in the axial plane

**Fig. 3:**
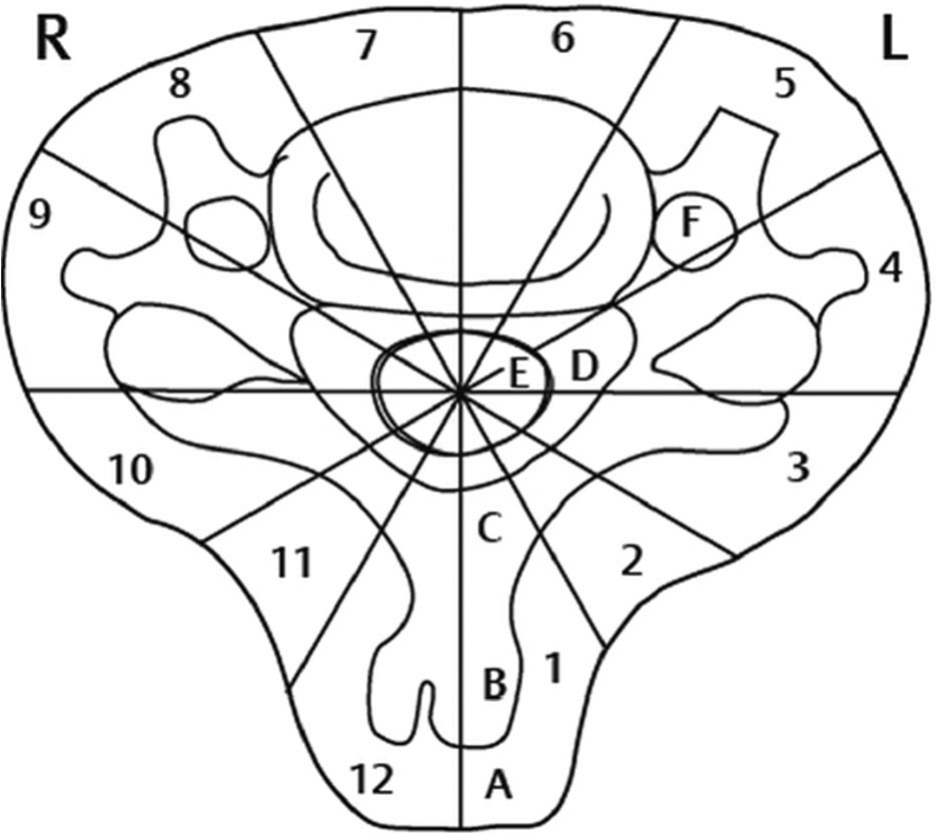
Weinstein, Boriani, Biagini (WBB) classification is divided into 12 sections with the central cord as the center, in a clockwise direction, starting from the spinous processes. The first region is the spinous process, the sixth region is the anterior vertebral corpus, and the 12th region is the right spinous process: (A) surrounding soft tissue, (B) intraosseous, (C) involving the vertebral canal, (D) located in the epidural space, (E) dura involvement, and (F) vertebral artery involvement

**Fig. 4:**
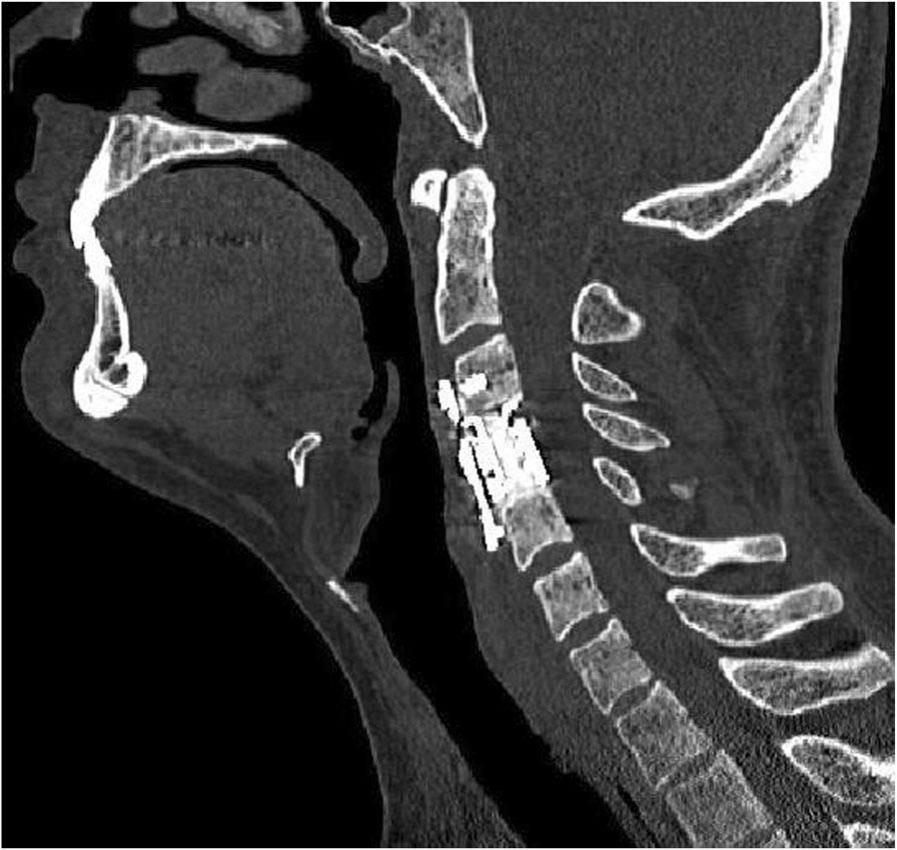
C4 corpectomy, corpectomy cage, and C3–5 anterior plate-screw image on postoperative CT in the sagittal plane

**Fig. 5:**
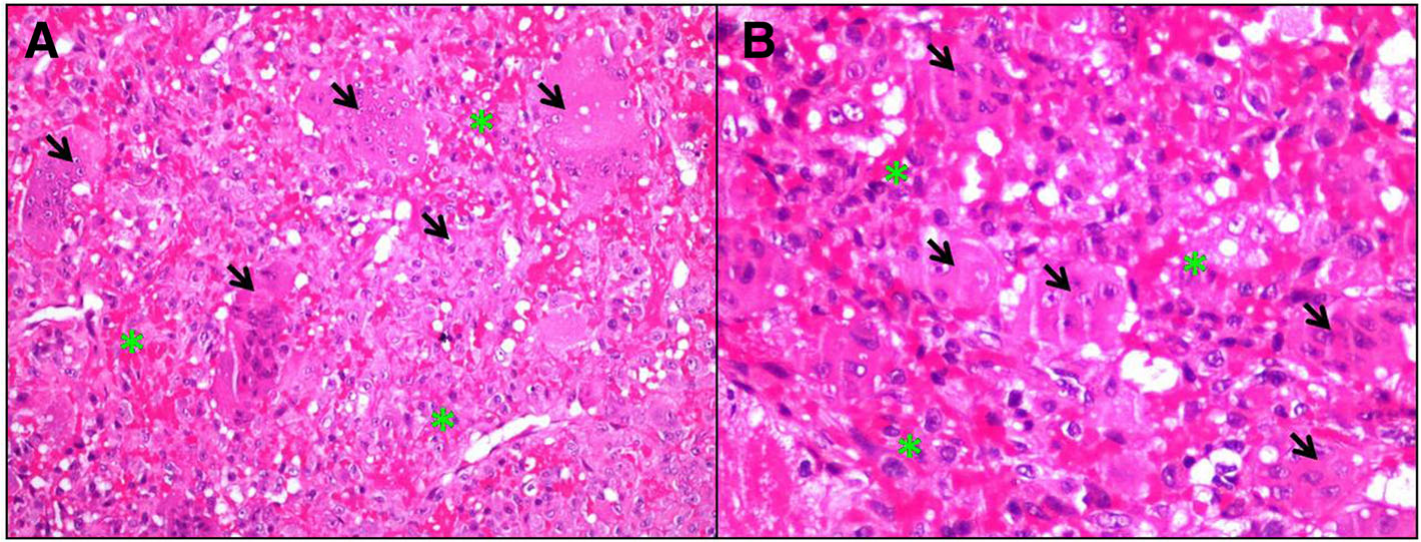
**a**, **b** Pathology figure. Osteoclastic multinuclear giant cells (arrowheads) interspersed in a stroma composed of cells with oval-fusiform nuclei (stars). (Hematoxylin and eosin, × 200, × 400 magnification, respectively)

**Fig. 6:**
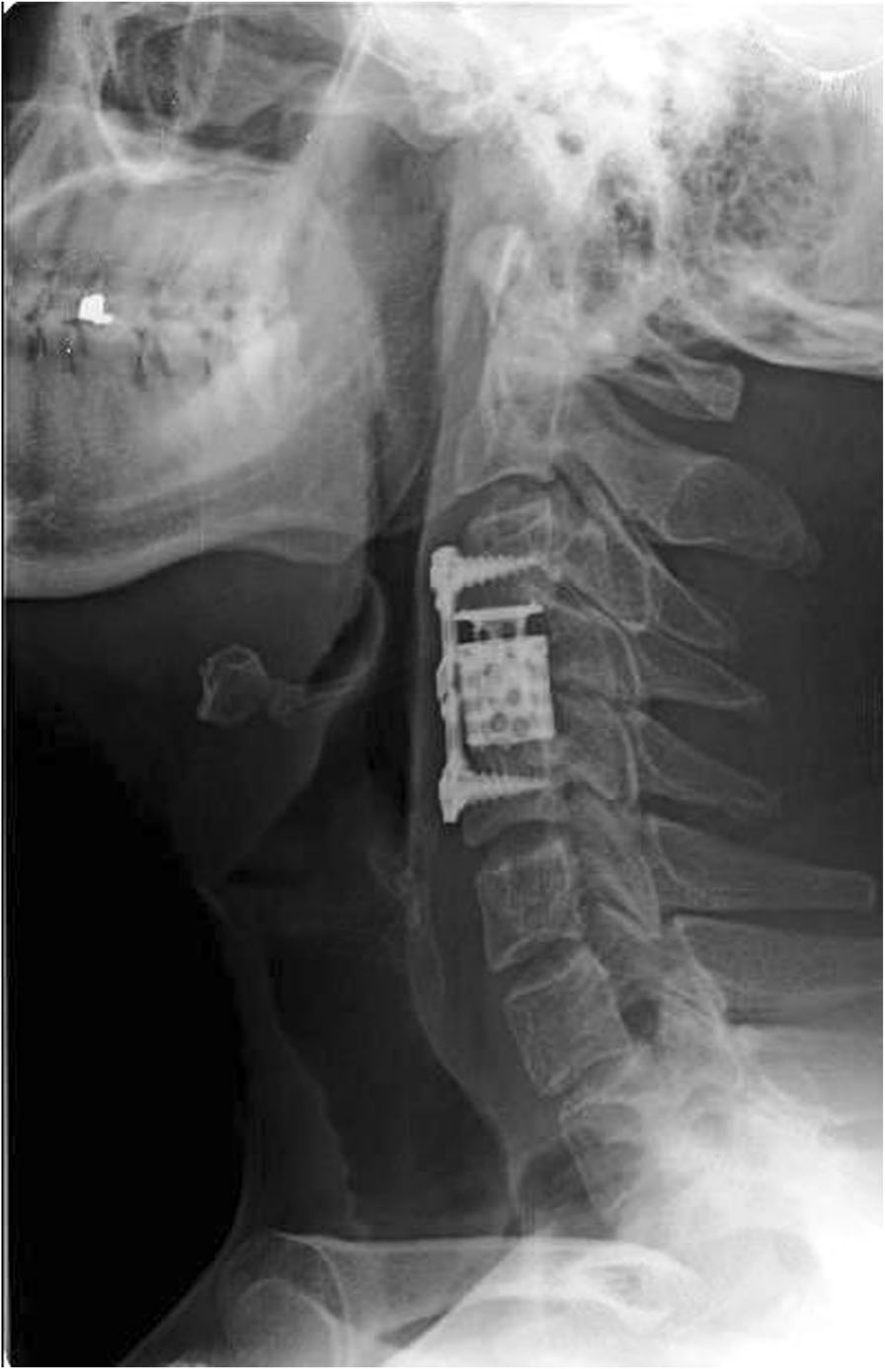
No recurrence was seen on the direct X-ray taken in the postoperative third year

A 31-year-old male patient presented to our clinic with symptoms of neck pain, back pain (pain in the lower cervical and upper thoracic region), and numbness in both arms for the last 3 months. His physical examination revealed hypoesthesia at the C4 and C5 dermatomes in both arms with no loss of strength. Cervical computed tomography (CT) showed a destructive and compressive lesion in the C4 vertebra corpus (Fig. 1). The retropulsion caused by compression had narrowed the canal. The lesion was also seen to be completely wrapped around the vertebral foramen at the right C4 level and to extend to the lateral mass posteriorly in the axial sections on CT (Fig. 2). Weinstein, Boriani, Biagini (WBB) classification was used for the classification of the tumor (Fig. 3) [1]. In this case, the tumor was located at the regions 5, 6, 7, 8, and 9 and invaded all the layers except the dura mater. Corpectomy was performed to the C4 vertebra with an anterior approach together with discectomy to the upper and lower disc spaces during surgery. The lesion was seen to extend to the right C4 vertebral foramen in the surgical observation after corpectomy, and the tumor was carefully dissected 360° around the vertebral artery at this level. Once the vertebral artery was revealed, we entered between the mass extending posteriorly to the lateral mass, the spinal cord, and the vertebral artery and performed meticulous intracavitary curettage. In order to ensure stability after tumor excision, the upper and lower corpus endplates were decorticated with the curette. A corpectomy cage was placed into the C4 space, and the system was fixed by placing a plate screw on the upper and lower vertebra from the anterior (Fig. 4). There was no additional neurological deficit postoperatively. The patient’s neurological complaints improved during the postoperative period. There was no residual or remaining tumor after resection. The pathological microscopical evaluation revealed a tumor rich in osteoclastic multinuclear giant cells interspersed in a stroma composed of cells with oval-fusiform nuclei. The pathological diagnosis was giant cell tumor of the bone (Fig. 5a, b). No recurrence was seen during 3 years of follow-up (Fig. 6).

## Discussion

GCTs are rare tumors and are quite rare in the cervical spine. Various treatment options such as surgery, radiotherapy, embolization, cryotherapy, and chemical adjuvants are used for spinal GCTs. Denosumab has been used in adjuvant therapy in recent years [9]. The aim of the treatment is to remove the tumor and prevent its recurrence while avoiding neurological structure damage and spinal integrity deterioration [10–13]. Although total en bloc resection is the best treatment method, it may not be possible as in other long bones due to reasons such as spinal cord injury during surgery, large vessel injury (thoracic aorta in the upper thoracal region, between T1–T4, ductus thoracicus and vertebral artery in the cervical region) due to blunt dissection, excessive bleeding, development of instability due to spinal osteotomies, and contamination during removal of the tumor cells, especially in the peduncle [14, 15]. Good results were reported with en bloc resection to decrease local recurrence in vertebral tumors by Boriani et al. [16]. Martin et al. recommended preoperative embolization followed by lesion resection for big lesions and en bloc resection in appropriate cases [10]. Although Marcove et al. [17] reported good results with cryotherapy, Leggon et al. [18] encountered a high local recurrence rate of 62% with cryotherapy followed by curettage. Radiotherapy (RT) is an option to decrease postoperative recurrence in GCTs. However, discussion continues on the development of myelopathy and sarcoma due to radiotherapy [19, 20]. Yang et al. reported sarcomatous changes in one of their three cases following postoperative RT [13]. RT should therefore mostly be reserved for recurring lesions [8, 13].

Curettage can be used for small lesions limited to the anterior cervical column, and anterior stabilization can be used for lesions limited to the vertebral corpus in cervical spine GCTs [11, 21, 22]. Surgery is performed in two stages as anterior and posterior for cases undergoing large excision. Anterior and posterior fusion is used to prevent instability after the excision [21, 23].

It is difficult to predict the prognosis in GCT cases as the recurrence rate is 11–50% even with the best treatment method of en bloc resection [8, 10–12, 24]. Local recurrences are most commonly seen in the first 3 years [8]. The local recurrence rate is 22–42% for all cases, but spinal GCT recurrence rates are from small series as spinal involvement by this disorder is rare [11, 25]. There is also no definite treatment scheme. Although our case had a cervical spine lesion extending posteriorly from the corpus, the vertebral corpus lesion was removed en bloc with a pure anterior approach and the lateral mass extension was excised with intracavitary curettage. A second surgery was therefore not required. Since radiotherapy use in the postoperative period is controversial, we decided not to administer radiotherapy to the patient after consulting with the radiation oncology department.

## Conclusions

In this case, the posteriorly located lesion was widely curetted through an anterior approach in a single session. The patient did not experience any recurrence during the 3 years of follow-up.

## References

[1] Boriani S, Bandiera S, Casadei R, Boriani L, Donthineni R, Gasbarrini A, Pignotti E, Biagini R, Schwab JH (2012). Giant cell tumor of the mobile spine: a review of 49 cases. Spine.

[2] Junming M, Cheng Y, Dong C, Jianru X, Xinghai Y, Quan H, Wei Z, Mesong Y, Dapeng F, Wen Y, Bin N, Lianshun J, Huimin L (2008). Giant cell tumor of the cervical spine: a series of 22 cases and outcomes. Spine (Phila Pa 1976).

[3] Lorenzo ND, Delfini R, Ciappetta P, Cantore G, Fortuna A (1992). Primary tumors of the cervical spine: surgical experience with 38 cases. Surg Neurol.

[4] Shirakuni T, Tamaki N, Matsumoto S, Fujiwara M (1985). Giant cell tumor in cervical spine. Surg Neurol.

[5] Öztop F, Zileli M, Özer AF (2002). Omurga tümörlerinin patolojisi. Omurilik ve Omurga cerrahisi, cilt 2.

[6] Dahlin DC (1977). Giant-cell tumor of vertebrae above the sacrum : a review of 31 cases. Cancer.

[7] Kim S, Kim Y, You S, Kim S, Park I, Baik M (2003). The trend of treatment for giant cell tumors of the spine in recent years. Age.

[8] Fidler MW (2001). Surgical treatment of giant cell tumours of the thoracic and lumbar spine: report of nine patients. Eur Spine J.

[9] Nakazawa T, Inoue G, Imura T, Miyagi M, Saito W, Namba T, Shirasawa E, Uchida K, Takahira N, Takaso M (2016). Remarkable regression of a giant cell tumor of the cervical spine treated conservatively with denosumab: a case report. Int J Surg Case Rep.

[10] Martin C, McCarthy EF (2010). Giant cell tumor of the sacrum and spine: series of 23 cases and a review of the literature. Iowa Orthop J.

[11] Sanjay BK, Sim FH, Unni KK, McLeod RA, Klassen RA (1993). Giant-cell tumours of the spine. J Bone Joint Surg Br.

[12] Thangaraj R, Grimer RJ, Carter SR, Stirling AJ, Spilsbury J, Spooner D (2010). Giant cell tumour of the spine sacrum: a suggested algorithm for treatment. Eur Spine J.

[13] Yang SC, Chen LH, Fu TS, Lai PL, Niu CC, Chen WJ (2006). Surgical treatment for giant cell tumor of the thoracolumbar spine. Chang Gung Med J.

[14] Su YP, Chen WM, Chen TH (2004). Giant-cell tumors of bone: an analysis of 87 cases. Int Orthop.

[15] Toribatake Y (1993). The effect of total en bloc spondylectomy on spinal cord circulation. J Jpn Orthop Assoc.

[16] Boriani S, Biagini R, De Iure F, Di Fiore M, Gamberini G, Zanoni A (1994). Lumbar vertebrectomy for the treatment of bone tumors: surgical technique. Chir Organi Mov.

[17] Marcove Ralph C., Sheth Dhiren S., Brien Earl W., Huvos Andrew G., Healey John H. (1994). Conservative surgery for giant cell tumors of the sacrum. The role of cryosurgery as a supplement to curettage and partial excision. Cancer.

[18] Leggon Robert E, Zlotecki Robert, Reith John, Scarborough Mark T (2004). Giant Cell Tumor of the Pelvis and Sacrum. Clinical Orthopaedics and Related Research.

[19] Feigenberg SJ, Marcus RB, Zlotecki RA, Scarborough MT, Berrey BH, Enneking WF (2003). Radiation therapy for giant cell tumors of bone. Clin Orthop Relat Res.

[20] Miszczyk L, Wydmanski J, Spindel J (2001). Efficacy of radiotherapy for giant cell tumor of bone: given either postoperatively or as sole treatment. Int J Radiat Oncol BioI Phys.

[21] Mattei TA, Ramos E, Rehman AA, Shaw A, Patel SR, Mendel E (2014). Sustained long-term complete regression of a giant cell tumor of the spine after treatment with denosumab. Spine J.

[22] Chen G, Li J, Li X, Fan H, Guo Z, Wang Z (2015). Giant cell tumor of axial vertebra: surgical experience of five cases and a review of the literature. World J Surg Oncol.

[23] Yoshioka K, Kawahara N, Murakami H, Demura S, Kawaguchi M, Oda M, Matsumoto I, Tomita K (2009). Cervicothoracic giant cell tumor expanding into the superior mediastinum: total excision by combined anterior-posterior approach. Orthopedics.

[24] Zileli M, Isik HS, Ogut FE, Is M, Cagli S, Calli C (2013). Aneurysmal bone cysts of the spine. Eur Spine J.

[25] Xu W, Li X, Huang W, Wang Y, Han S, Chen S, Xu L, Yang X, Liu T, Xiao J (2013). Factors affecting prognosis of patients in a single center. Ann Surg Oncol.

